# Proteomic profile of Laser-dissected Motoneurons, Ependymal Cell Layer and Dorsal Root Ganglia after Spinal Cord Injury in the Rat

**DOI:** 10.1038/s41597-026-07125-2

**Published:** 2026-04-03

**Authors:** Olga Gajewska-Woźniak, Agata Pytyś, Tomasz Wójtowicz, Remigiusz Serwa, Kasia Radwanska, Małgorzata Skup

**Affiliations:** 1 Nencki Institute of Experimental Biology, Polish Academy of Sciences, 3 Pasteur St., Warsaw, 02-093 Poland; 2 Proteomics Core Facility, International Institute of Molecular Mechanisms and Machines Polish Academy of Sciences, Warsaw, Poland

## Abstract

Spinal cord injury induces profound molecular changes in surrounding tissue. Deciphering these changes with cell type-specific resolution shall facilitate discovery of new molecular targets that promote recovery. Here, we performed a proteomic analysis of laser-dissected motoneurons (MNs), ependymal cells (ECs), and dorsal root ganglia (DRG) obtained from adult rats 2 or 6 weeks after thoracic spinal cord transection at T11-T12 level, and control animals (n = 5-6 rats per group). We traced with fluorescent cholera toxin and laser-microdissected MNs innervating soleus muscle (SOL, n = 172 ± 39 MNs sections per animal, mean ± SD) and tibialis anterior muscle (TA, n = 262 ± 74 per animal) at lumbar spinal cord segments L3–L5. The ECs and DRG were dissected from the same lumbar segments. Mass spectrometry analysis of the samples allowed us to detect 1221 proteins in SOL MNs, 1186 in TA MNs, 1520 in ECs layer, and 5087, 3740 and 3086 in DRG L3, L4 and L5, respectively. Here we describe how this data was obtained and made available for further use. Our data may help to characterize molecular mechanisms regulated in the rat spinal MNs, DRG and ECs in the early and late period after spinal cord transection.

## Background & Summary

Spinal cord injuries (SCI) remain a critical health and societal challenge worldwide, leading to severe and often irreversible functional impairments. Despite significant advances in therapeutic approaches, including epidural stimulation and treatment with biomaterials with cell-based systems, many obstacles to effective recovery persist^1,2^. A growing body of evidence highlights that motoneurons (MNs) innervating distinct muscle groups possess unique physiological and molecular characteristics. In particular, we recently demonstrated that SCI in rats differentially alters protein and mRNA levels of neurotransmitter and neurotrophin receptors in tracer-identified MNs innervating ankle joint extensor (soleus, SOL and gastrocnemius lateralis, GL) and flexor (tibialis anterior, TA) muscles^3–7^. However, detailed proteomic profiles of these MNs are lacking. Deciphering the proteomic heterogeneity of MNs innervating different muscles, as well as their adaptive responses to injury and stimulation, is crucial for identifying novel therapeutic targets and strategies of precision medicine. However, significant methodological challenges, such as reliable distinguishing and isolating homogeneous populations of MNs, remain. While amyotrophic lateral sclerosis (ALS) studies have used fluorescence-activated cell sorting (FACS) of cultured neurons^8^ or laser microdissection (LMD)^9,10^ to isolate unidentified MNs from murine and human spinal cords for mass spectrometry analyses, so far no study has examined proteomic profiles of target-defined MNs in healthy and diseased state after SCI.

To address this gap, we performed proteomic profiling of retrogradely labeled MNs isolated by laser microdissection (LMD) from lumbar spinal segments L3–L5. We focused on MN pools innervating two antagonistic hindlimb muscles, the SOL and TA which play complementary roles in locomotion by controlling ankle extension and flexion, respectively. These pools differ in motor unit physiology^11,12^ and activity profiles, with SOL MNs predominantly tonically active in postural control and TA MNs phasically active during locomotion^13,14^. They also exhibit distinct disease vulnerability^15^ and injury-induced plasticity. Our previous work showed that, unlike MNs innervating TA, MNs supplying SOL and its synergist GL undergo pronounced loss of synapses after spinal cord transection, accompanied by differential receptor responses^5–7,16^. These distinct motor pools provide a suitable model to identify motor pool -specific proteomic signatures.

Complete spinal cord transection (SCT) at the thoracic T11–T12 spinal cord segments, results in complete paralysis of the hindlimbs while preserving respiratory and essential autonomic functions. Lumbar segments caudal to the lesion remain structurally preserved and undergo spontaneous remodeling in the absence of descending supraspinal inputs^17^. Therefore, this region represents a promising target for therapeutic interventions aimed at restoring locomotor functions.

Proteomic analyses were conducted at early (2 weeks) and chronic (6 weeks) post-injury time points to capture both subacute injury responses and longer-term remodeling processes relevant to therapeutic intervention strategies.

In addition, we performed proteomic analysis of DRG from L3, L4, L5 lumbar segments, which house the cell bodies of sensory neurons that transmit peripheral information to the central nervous system, including monosynaptic inputs to MNs. These ganglia play a pivotal role in transmitting and perception of pain and other sensory modalities, which are often altered after SCI.

Finally, we included in the study ependymal cells (ECs) lining the spinal cord’s central canal, which contribute to cerebrospinal fluid (CSF) production and distribution. In the light of data showing the stimulatory effect of injury on ECs proliferation, we expected that their proteome undergoes remodeling^18^. ECs have been proposed as adult neural stem cells with therapeutic potential for spinal cord repair. Although their regenerative capacity was reported as limited and localized^19^, analyzing proteomic changes can confirm their phenotypic adaptations.

Together, this study offers a comprehensive proteomic dataset obtained from target-defined MNs, DRGs and ECs, key cell types involved in motor and sensory function and regeneration after spinal cord injury, paving the way for more targeted and effective therapies.

## Materials & Methods

### Animals

Experiments were conducted on adult male Wistar rats (body weight: 270–325 g at the start of the study), bred and housed at the Nencki Institute of Experimental Biology PAS in Warsaw, Poland. Animals had free access to standard pellet food and water and were maintained on a 12-hour light/dark cycle, in groups of 4–6. All experimental procedures, including surgeries and postoperative care, were approved by the 1st Local Ethics Committee in Warsaw (decision numbers 1481P1/2023 and 1482P2/2023), in accordance with Directive 2010/63/EU of the European Parliament and Council on the protection of animals used for scientific purposes. All methods followed the ARRIVE guidelines.

The main experiment was carried out on 16 rats. Fluorescent retrograde tracers were injected into hindlimb muscles, the SOL and TA. One week later in 10 animals, spinal cord transection (SCT) was performed at the thoracic T11-T12 spinal cord level. Five rats were sacrificed 2 weeks post-SCT (SCT2W group, n = 5), and five at 6 weeks post-SCT (SCT6W, n = 5). Control rats (n = 6) were sacrificed 2 weeks after tracer injection. Coded animal IDs were used; codes were revealed after analysis.

### Retrograde tracing of motoneurons

As in our previous studies^6,16,20^, all animals were premedicated with subcutaneous Butomidor (butorphanol, 1.5 mg/300 g body weight; Richter Pharma, Wels, Austria) and anesthetized with isoflurane (1–2.5% in oxygen; Baxter, Lessines, Belgium) delivered via a face mask.

**Fig. 1:**
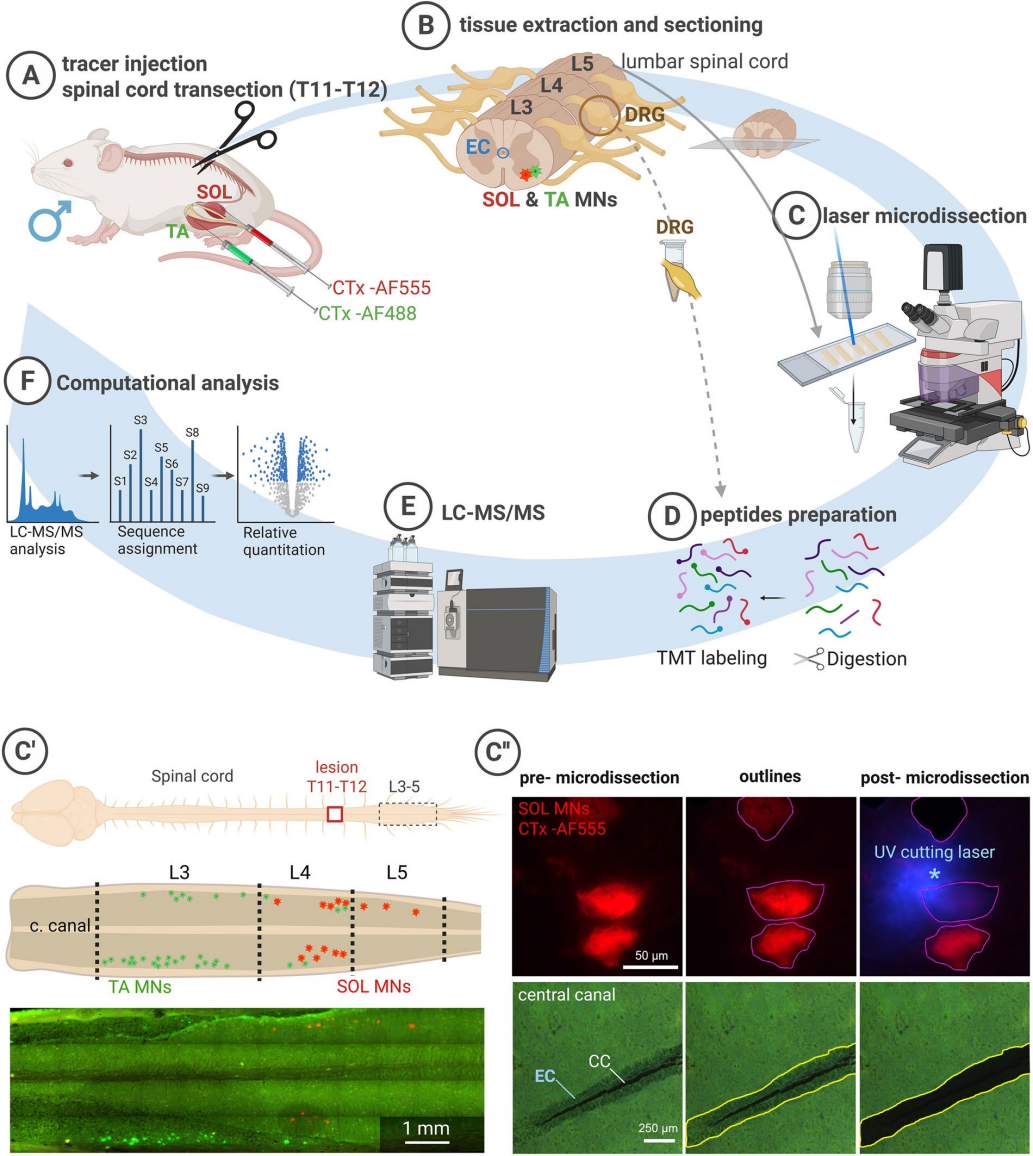
Experimental design and proteomic workflow. (**A**) To label soleus (SOL) and tibialis anterior (TA) motoneurons (MNs), adult Wistar rats (n = 16) received injections of retrograde CTx tracers into SOL and TA muscles. (**B**) Rats were sacrificed 2 (SCT2W, n = 5) or 6 weeks (SCT6W, n = 5) after SCT; six rats served as healthy controls. (**C**) Laser microdissection (Leica LMD7000) system was used to isolate cells from spinal cord slices. **C’**. A schematic drawing of the lesion site located at thoracic T11-T12 level, and analyzed lumbar L3–L6 segments showing distribution of SOL (red) and TA (green) MNs (top), and representative fluorescence microphotograph of snap-frozen longitudinal spinal cord section with retrogradely labeled MNs (bottom). Note that the imaged tissue to be microdissected was not PFA-fixed and covered with mounting medium or coverslips; therefore, image quality is suboptimal. In rats, the estimated number of SOL MNs is ~60 per leg and TA MNs is ~130 per leg. All visible TA and SOL MSs and ECs (L3-L5) were microdissected. **C”**. Fluorescence microphotographs of MNs and ECs pre- and post- laser microdissection. (**D**) Proteins were extracted, digested, and labeled with tandem mass tags (TMT). (**E**) Peptides were off-line fractionated at high pH and then separated using an Easy-Spray PepMap column on an UltiMate 3000 nano-LC system coupled to a Q Exactive HF-X mass spectrometer. (**F**) Data was processed in MaxQuant (v1.6.17.0) with identification via the Andromeda search engine against the *Rattus norvegicus* UniProt database. Figure generated with Biorender.

The skin over the SOL and TA muscles was shaved and cleaned using 70% ethanol. To retrogradely label MNs innervating these muscles, cholera toxin subunit B (CTx) conjugated with Alexa Fluor 555 or Alexa Fluor 488 (0.01% in phosphate-buffered saline; Molecular Probes, USA) was bilaterally injected into the respective muscles. Into each SOL muscle 10 µl of CTx AF555 (red) were injected using a Hamilton microsyringe equipped with a 26-gauge needle and to each TA muscle 20 µl of CTx AF488 (green) were injected, using a Hamilton microsyringe with a 22-gauge needle (Fig. 1A).

Injection sites were chosen based on prior motor endplate mapping^21,22^. Multiple injection sites and needle penetration of muscle were used. Occasional muscle twitching during injection served as confirmation that the needle was in the proper location. Each injection lasted approximately 10 minutes. To minimize tracer leakage, the needle was left in place for 3 minutes following injection. Afterward, the site was rinsed with sterile saline and the skin was sutured.

Postoperative care included subcutaneous administration of Tolfedine (tolfenamic acid 4%, 4 mg/kg; Vetoquinol, Lure Cedex, France) for analgesia over five days, and Baytril (enrofloxacin, 5 mg/kg; Bayer GmbH, Leverkusen, Germany) once daily for five consecutive days to prevent infection. After recovery from anesthesia, animals were returned to their home cages with *ad libitum* access to food and water.

### Spinal cord transection

Approximately one week after tracer administration, rats from the SCT2W (n = 5) and SCT6W (n = 5) groups underwent complete SCT. Surgical procedures (Fig. 1A) were performed as previously described^23^. SCT at the T9 vertebral level, corresponding to the T11–T12 spinal cord segments, results in paralysis of the hindlimbs.

Briefly, the dorsal skin was shaved and cleaned with 70% ethanol, then incised at the level of the lower thoracic vertebrae. Muscles and ligaments were carefully separated to expose the vertebrae. Following identification of the T9 vertebral level, a laminectomy was performed. The dura mater was opened, and 2% lidocaine (Lignocainum hydrochloricum; Polfa Warszawa S.A., Poland) was applied topically to the spinal cord before it was completely transected using fine surgical scissors. The lesion gap was then gently enlarged to approximately 0.5 mm by aspiration, and the site was rinsed with a 0.9% NaCl solution. The lesion area was carefully inspected under a surgical microscope (Nikon SMZ 1000) to confirm completeness of transection. Surrounding tissues were repositioned, and the muscle and skin layers were sutured.

Following surgery, approximately 5 ml of 0.9% NaCl was administered subcutaneously for rehydration. Postoperative care included subcutaneous administration of the antibiotic Sultridin (30 mg/kg; Norbrook, Ireland) once daily for five consecutive days, and the analgesic Vetaflunix (2.5 mg/kg; VET AGRO, Poland) for three days.

Immediately post-surgery, each animal was placed in a clean recovery cage on a heated mat and monitored until fully awake (approximately 1 hour), after which they were returned to their home cages with free access to food and water.

Animals were monitored three times daily during the first postoperative week, and twice daily during the second week. Care included general health inspection, cleaning of the perineal area, and manual bladder expression when necessary. Spontaneous micturition typically resumed during the second postoperative week. No major health complications were observed in any animal throughout the duration of the experiment.

The SCT is a well-established and highly reproducible model. Consistent with our previous studies, SCT rats exhibit paraplegia, with BBB scores remaining in the 0–2 range at six weeks post-injury, indicating only occasional, non–weight-bearing hindlimb movements. Sensory function of the hindpaws assessed with von Frey filaments typically shows mechanical withdrawal thresholds of 18.75 ± 3.20 g (mean ± SEM) in intact animals, 13.38 ± 2.03 g at 6–9 days after spinalization, and 10.14 ± 1.92 g at 30 days post-injury, based on our earlier cohorts. Thresholds decrease over time, consistent with the development of spinal hyper-reflexia and increased reflex excitability following loss of supraspinal control^24^. Behavioral and locomotor outcomes after SCT have been reported previously by our group^6,25^.

### Tissue preparation and laser microdissection (LMD)

Animals were anesthetized with isoflurane and euthanized by a lethal intraperitoneal injection of pentobarbital (120 mg/kg body weight; Morbital, Biowet, Poland).

Rats were then transcardially perfused with 250 ml of ice-cold 0.01 M phosphate-buffered saline (PBS: 154 mM NaCl, 1.3 mM Na₂HPO₄, 2.5 mM NaH₂PO₄; pH 7.4). The vertebral column was excised and placed on ice. Lumbar spinal cord segments (L3–L6, Fig. 1B), approximately 1.2 cm long, were rapidly dissected, snap-frozen in dry ice-cooled tubes, and stored at −80 °C until further processing (within one month). Dorsal root ganglia (DRGs) from segments L3, L4, and L5 were dissected bilaterally as whole structures. The L3 and L4 DRGs are the largest ganglia within the lumbar region and can be easily distinguished based on their size and anatomical location. Left and right ganglia from each lumbar level were pooled and frozen. Frozen L3–L6 spinal cord segments were embedded in Jung tissue-freezing medium (Leica, cat. no. 14020108926) and longitudinal sections were cut horizontally at 20 µm thickness using a cryostat (Slee MEV, SLEE Medical GmbH) at −20 °C. Sections were mounted onto RNase-free PEN membrane frame slides (Leica No. 11505190 or Applied Biosystems™ LCM0521), with 6–9 sections per slide and approximately 3 slides per animal. Slides were stored on dry ice or at −80 °C for up to one week prior to microdissection.

Prior to microdissection, slides were dehydrated through a graded ethanol series (70%, 80%, 90%, and 2 × 100%, each for 30 s), followed by two xylene washes (30 s and 180 s), and air-dried for 3–5 minutes.

MNs and ECs tissue samples were isolated using the Leica LMD7000 Laser Microdissection System. PEN membrane frame slides were placed in the slide holder, and RNase-free 0.2 ml tube caps were positioned in tube holders for gravity-based sample collection. Labeled MNs (SOL with CTx-Alexa Fluor 555, and TA with CTx-Alexa 488) were visualized under the microscope using first 10 × and then 40 × (NA 0.6) objectives, and individually outlined and dissected by a UV laser (Fig. 1B). Laser parameters were preset for each objective and further adjusted to minimize laser power while maintaining efficient tissue cutting. For the 40 × objective, with a maximal pulse energy of 120 µJ, the settings (relative units) were: laser power 34, aperture 12–15, cutting speed 10–24, and pulse frequency 120.

ECs were identified as a single-layered lining of the central canal (surrounding the spinal cord’ central lumen) and the autofluorescence of the surrounding tissue (Fig. 1C**”**) in the same tissue sections as MNs. Due to their small size (6–8 µm) and dense arrangement, ECs were microdissected as a continuous layer using the 10 × objective (NA 0.32). The settings were: laser power 34–42, aperture 5–8, cutting speed 10–20 and pulse frequency 910–1046.

Cut samples were captured directly into the tube caps by gravity. Following sample collection from each membrane slide (typically up to 3 hours/slide), tubes were sealed and stored on dry ice or at −80 °C (inverted, cap-down) until the next step.

### Sample preparation

Samples were processed following a modified trifluoroacetic acid (TFA)-based protocol^26^. Tissue-containing caps from laser microdissection were supplemented with trifluoroacetic acid (≥99% TFA (302031, Sigma Aldrich), 10 μL for MN and ECs samples, 40 μL for DRG L3, L4, and 30 μL for DRG L5 samples. Samples were mixed by shaking for 3 min, followed by brief centrifugation and 1 min of sonication in an ultrasonic water bath. Following centrifugation, each sample was neutralized by adding a 10-fold volume of 2 M Tris (T1503, Sigma Aldrich) buffer (pH 8.5). Subsequently, a reduction/alkylation buffer - containing 100 mM tris(2-carboxyethyl)phosphine, TCEP (75259, Sigma Aldrich) and 400 mM CAA (2-chloroacetamide, C0267, Sigma Aldrich) - was added in a volume equal to 1.1 × that of the original TFA volume. Samples were incubated at 95 °C for 5 min. Protein digestion was carried out using sequencing grade modified trypsin (V5111, Promega) at 37 °C overnight. Digestion was halted by the addition of TFA to a final concentration of 1% v/v. Tryptic peptides were labeled using an on-column tandem mass tag (TMT) labeling protocol^27^. Individual TMT-labeled peptide samples were pooled into multiplex samples and concentrated using a SpeedVac concentrator. The multiplex peptide samples were fractionated (6 fractions) using Pierce™ High pH Reversed-Phase Peptide Fractionation Kit (84868, Thermo Fisher Scientific).

### Liquid chromatography–mass spectrometry (LC-MS/MS) measurement

Mass spectrometry analysis was performed in the Proteomics Core Facility, International Institute of Molecular Mechanisms and Machines Polish Academy of Sciences (IMol PAS), Warsaw, Poland. Peptide fractions were resuspended in 0.1% trifluoroacetic acid (TFA) and LC-MS grade 2% acetonitrile (1.00029, Supelco) in water (1.15333, Supelco) prior to analysis. Chromatographic separation was carried out using an Easy-Spray Acclaim PepMap column (50 cm × 75 µm ID; PN ES903, Thermo Fisher Scientific) maintained at 55 °C. Peptides were eluted over a 90-minute gradient of acetonitrile in 0.1% aqueous formic acid at a flow rate of 300 nL/min using an UltiMate 3000 nano-LC system (Thermo Fisher Scientific). The LC system was coupled via an Easy-Spray ion source to a Q Exactive HF-X Orbitrap mass spectrometer (Thermo Fisher Scientific), operating in TMT mode. Full MS (survey) scans were acquired at a resolution of 60,000 at m/z 200. Up to 15 of the most abundant isotope patterns with charges 2–5 were selected for MS/MS fragmentation using higher-energy collision dissociation (HCD) with a normalized collision energy (NCE) of 32. Precursor ions were isolated with a 0.7 m/z window, and a dynamic exclusion of 35 seconds was applied. The maximum injection times were set to 50 ms for MS and 150 ms for MS/MS scans. MS/MS spectra were acquired at a resolution of 45,000 (at m/z 200). The automatic gain control (AGC) target values were 3e6 for MS and 1e5 for MS/MS, with a minimum AGC target of 1e3.

### LC-MS/MS data processing

Raw MS data files were processed using MaxQuant software (version 1.6.17.0), with peptide identification performed via the built-in Andromeda search engine^28^. Spectra were searched against the UniProt *Rattus norvegicus* reference proteome (UP000002494). Reporter ion MS2-based quantification was employed with a reporter mass tolerance of 0.003 Da and a minimum reporter ion purity (PIF) threshold of 0.75. Carbamidomethylation of cysteines was set as a fixed modification, while oxidation of methionine, deamidation of asparagine/glutamine, and N-terminal acetylation were set as variable modifications. Protein digestion was simulated with trypsin/P specificity (cleavage after lysine or arginine, including before proline), allowing for up to two missed cleavages. False discovery rates (FDR) were set at 1% (0.01) for peptides, proteins, and modification sites. The “match between runs” feature was enabled to increase peptide identification across samples. Other parameters were used at default settings. Reporter intensity-corrected values for protein groups were imported into Perseus (version 1.6.10) for statistical analysis^28^. Standard filtering steps were applied to remove: reverse hits (from decoy database), proteins identified only by modification site, common contaminants (based on MaxQuant’s internal list). Reporter intensities were log₂-transformed, and only protein groups quantified across all samples were retained. Data were normalized by median subtraction within TMT channels to correct for systematic variation. Each biological replicate (i.e., each animal) was processed independently, and for each animal every sample type generated a separate proteomic dataset. For downstream statistical analyses, datasets from the same sample type (e.g., SOL MNs, TA MNs, ECs) were grouped across animals and treated as biological replicates. Differential expression analysis was performed using two-sided Student’s t-tests with significance thresholds -log10p-value ≥ 1.3 (p-value ≤ 0.05) and −0.4 ≥ log2FC ≥ 0.4 (fold change ≈ 1.32). The final statistical tables were exported from Perseus and formatted using Microsoft Excel 2016.

## Data Records

The mass spectrometry proteomics data have been deposited to the ProteomeXchange Consortium^29^ via the PRIDE^30^ partner repository with the dataset identifier PXD070428^31^. The submission includes 36 raw LC–MS/MS files (6 sets, 6 fractions each), MaxQuant outputs (three txt folders corresponding to three independent processing runs: sets 1–2, MN samples, processed together; set 3, ECs of the central canal, processed separately; and sets 4–6, DRG, processed together), and documentation describing the six TMT-multiplexed sample sets. Alternatively, tables with processed data and comparisons between sample groups were deposited at RepOD (Open Data Repository, 10.18150/9VXNRH)^32^. These tables include normalized protein intensities, differential abundance analyses, and functional annotations, complementing the PRIDE raw data with user-ready, comparative results.

## Data Overview

The Table 1 reports the total number of proteins identified in each sample type using the analytical workflow described in the Methods section, as well as the number of proteins significantly up- or downregulated at 2 weeks (SCT2W) and 6 weeks (SCT6W) post-SCT relative to controls. Differentially expressed proteins between 2 and 6 weeks post-injury are included.

**Table 1:** Summary of protein identification and differential expression across analyzed cell types and time points following spinal cord transection (SCT).

Sample	number of detected proteins	number of up- or down- regulated proteins		
		SCT2W vs CTRL	SCT6W vs CTRL	SCT2W vs SCT6W
SOL MNs	1221	36	15	11
TA MNs	1186	11	4	5
Ependymal cells (EC)	1520	84	19	22
DRG L3	5087	24	88	19
DRG L4	3740	24	36	54
DRG L5	3086	59	31	12

## Technical Validation

### Optimization of neurotracing and lysis strategies for proteomic analysis of PEN-mounted cryosections

In preliminary experiments Fast Blue neurotracer (2% aqueous solution; Dr. Illing Plastics GmbH, Germany) to retrogradely label MNs was tested; due to rapid fluorescence fading and suboptimal motoneuron yield it was excluded from further use and was replaced successfully with a photostable fluorescent cholera toxin subunit B conjugate.

**Fig. 2:**
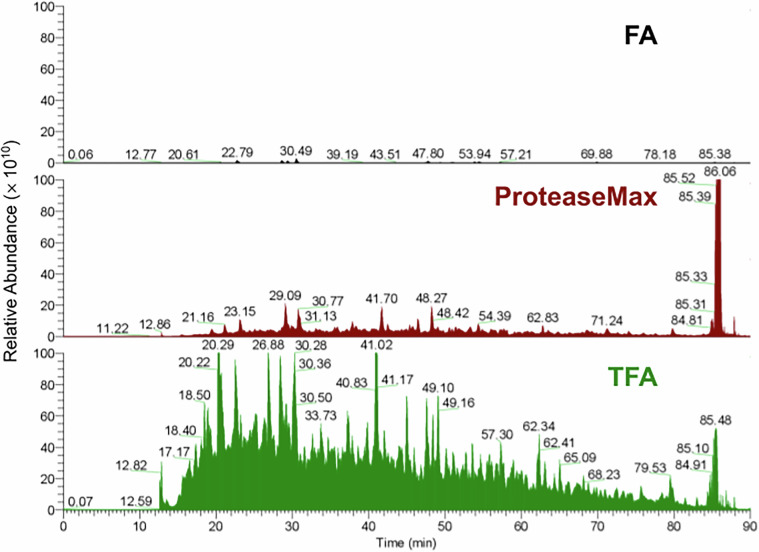
Comparison of protein extraction methods from frozen spinal cord sections mounted on PEN membranes. Representative total ion current (TIC) chromatograms after extraction with formic acid (FA; black), ProteaseMAX (red), or trifluoroacetic acid (TFA; green). TFA produced the highest TIC and yielded the greatest number of protein identifications relative to FA and ProteaseMAX. TIC intensity (y-axis) is plotted against chromatographic time (x-axis). Chromatograms were exported with Thermo Scientific Xcalibur Qual Browser (v4.5).

A pilot experiment was conducted to assess whether it is feasible to identify proteins in frozen tissue sections that were cut together with the fragments of PEN membrane onto which they were mounted. Several protein extraction methods were tested, including trifluoroacetic acid (TFA), formic acid (FA), and the ProteaseMAX surfactant reagent (V2071, Promega) in HEPES buffer (Fig. 2). The best results for small spinal cord samples (1 mm², 20 µm thickness) were obtained using TFA, which enabled the identification of 2 492 proteins. In comparison, the application of ProteaseMAX or FA allowed the identification of 2 054 and 618 proteins, respectively. It was also confirmed that the PEN membrane remains stable in acidic conditions and does not interfere with analytical results.

### Experimental reproducibility and design

Each experimental group (three conditions: sham control, 2 and 6 weeks post-SCT) included 5-6 biological replicates: control (n = 6), SCT2W (n = 5), SCT6W (n = 5). Final datasets were obtained for SOL and TA motoneurons: control, n = 5; SCT2W, n = 5; SCT6W, n = 5; for Ependymal cells: control, n = 5; SCT2W, n = 5; SCT6W, N = 5; for DRG L3 and L4: control, n = 4; SCT2W, n = 5; SCT6W, n = 5; for DRG L5: control, n = 3; SCT2W, n = 4; SCT6W, n = 5. Samples were analysed in 6 batches. To minimize inter-batch variation, samples of a given type (SOL MNs, TA MNs, EC, DRG L3, DRG L4, DRG L5) were analyzed within a single TMT 16-plex set/batch.

### Consistency of laser microdissection of motoneurons and ependymal cell layer

Retrogradely traced SOL and TA MNs, as well as ECs layers were laser microdissected. DRG L3-5 were dissected as whole structures (4–8 mg wet mass per left + right ganglion).

The average dissected tissue area (mean ± SD) per animal was 0.24 ± 0.058 mm² for SOL MNs, 0.34 ± 0.078 mm² for TA MNs, and 0.89 ± 0.038 mm² for ECs layer. Corresponding numbers of dissected MNs sections were 172 ± 39 for SOL, 262 ± 74 for TA, and 30 ± 18 fragments for ECs layer. The mean area of cut MN sections was relatively consistent across groups, with 1428 ± 101 μm² for SOL and 1 341 ± 220 μm^2^ for TA. This value was not determined for individual ECs because of their small dimensions (6–8 micrometers) and tight fit; they were cut as a layer.

In rats, the SOL motor pool contains approximately 60 MNs per hindlimb, whereas the TA motor pool contains approximately 130 MNs per hindlimb^33–35^. Considering that each MN soma (~40 µm in diameter, estimated from the mean MN cross-sectional area; Table 2) can appear in 2–3 consecutive tissue sections (20 µm thickness), we estimate that approximately 43 SOL MNs and 65 TA MNs per animal were isolated.

**Table 2:** Parameters of microdissected samples.

Averaged parameters of microdissected samples per animal ( ± SD)				
	number of MN sections/ECs fragments	mean area of cell [μm^2^]	total dissected area [mm^2^]	tissue mass* [ng]
**SOL MNs**	**172** ± **39**	**1428** ± **101** (Control: 1499 ± 51, SCT2W: 1376 ± 137, SCT6W: 1377 ± 74)	**0.24** ± **0.058**	**4.89** ± **1.16**
**TA MNs**	**262** ± **74**	**1341** ± **220** (Control: 1468 ± 243, SCT2W: 1143 ± 77, SCT6W: 1331 ± 133)	**0.34** ± **0.078**	**6.78** ± **1.56**
**ECs layer**	**30** ± **18**	—	**0.89** ± **0.038**	**16.39** ± **8.74**

*estimated.

The slightly lower efficiency of TA MNs isolation (25%) compared to SOL MNs (36%) may be associated with the more dispersed distribution of motor endplates in the TA muscle, potentially affecting tracer uptake, and with the positioning of TA MNs at the edges of tissue sections, where cells are more susceptible to sectioning-related damage.

Tissue volume was calculated by multiplying the dissected total area by the slice thickness (20 μm), and tissue mass was estimated assuming a brain tissue density of 1 g/cm³^36^. The estimated wet mass of sampled tissue was: 4.89 ± 1.16 ng for SOL MNs, 6.78 ± 1.56 ng for TA MNs, and 16.39 ± 8.74 ng for ECs (Table 2).

### Data quality and reproducibility

**Fig. 3:**
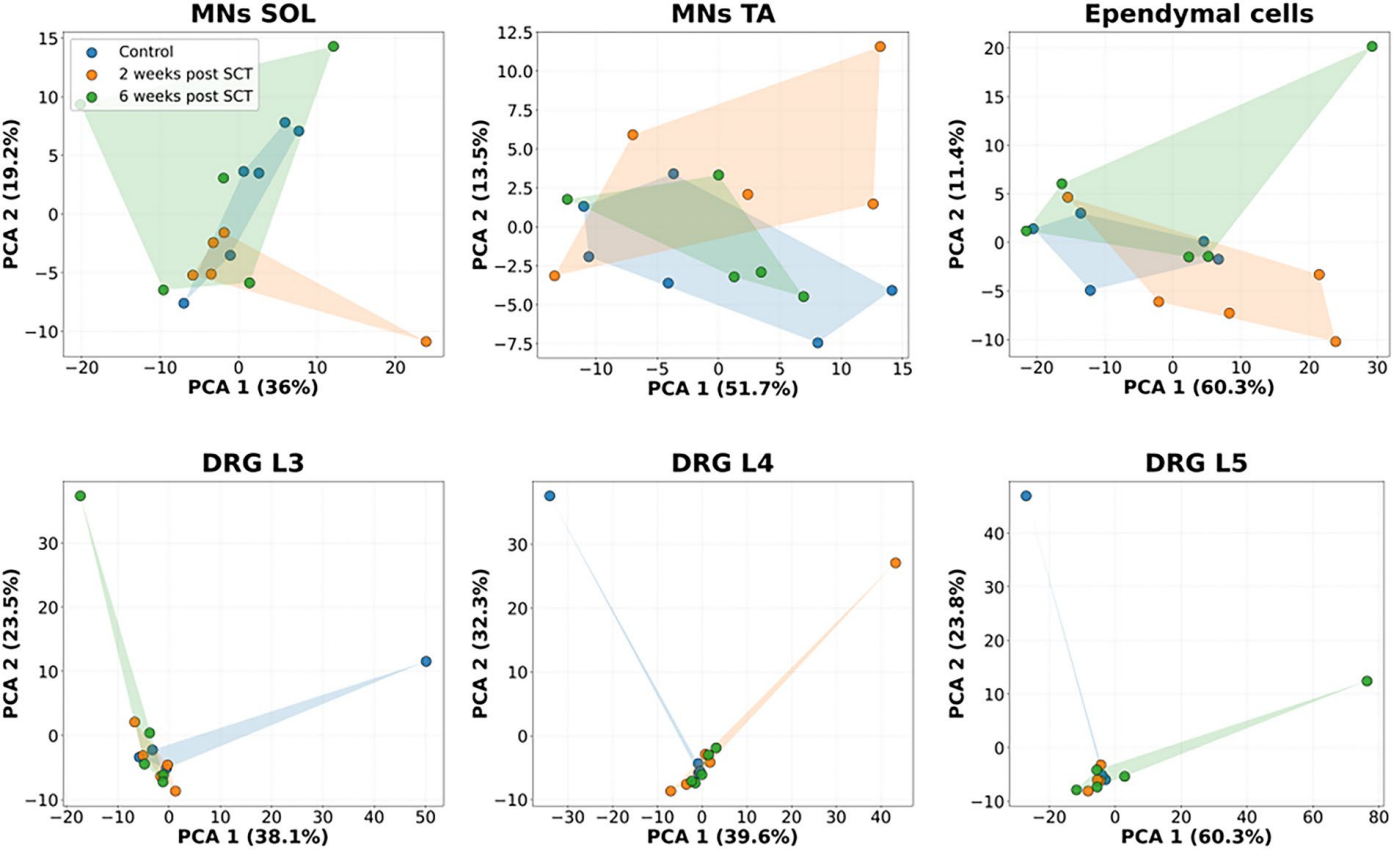
Principal component analysis (PCA) of proteomic profiles across all 6 sample types and 3 experimental groups. Each plot displays the distribution of biological replicates along PC1 and PC2 for: SOL and TA MNs, ependymal cells, and DRG from L3, L4, and L5 segments. Percent variance explained by each component is indicated on the corresponding axes.

Principal component analysis (PCA) was performed to assess the consistency and separation of proteomic profiles across 6 data sets (for each sample type) and 3 experimental groups (control, SCT2W, SCT6W). In motoneurons, the first two components explained 36.0% (PC1) and 19.2% (PC2) of the total variance for SOL MNs, and 51.7% (PC1) and 13.5% (PC2) for TA MNs, demonstrating good within-group homogeneity and clear distinction between subtypes (Fig. 3). In ependymal cells (EC), PCA captured 60.3% (PC1) and 11.4% (PC2) of the variance, indicating highly consistent expression patterns and a strong injury-associated signature. In dorsal root ganglia (DRG), variance explained by PC1 and PC2 ranged from 38.1%/23.5% (L3) and 39.6%/32.3% (L4) to 60.3%/23.8% (L5), reflecting robust reproducibility and distinct segmental differentiation. Overall, PCA confirmed high biological consistency within each sample type and separation between experimental conditions (Table 3).

**Table 3:** PCA analysis of 6 datasets from 3 experimental groups of Control, SCT2W and SCT6W rats.

Sample type	PC1 variance %	PC2 variance %	Interpretation
SOL MNs	36%	19.2%	Moderate variance capture; samples show good internal consistency and distinct but partly overlapping proteomic profiles across groups. Indicates heterogeneous responses after SCT.
TA MNs	51.7%	13.5%	High variance capture; clear separation between experimental groups and strong internal homogeneity.
Ependymal cells (ECs)	60.3%	11.4%	High explained variance; consistent proteomic profiles and pronounced injury-related clustering. Indicates a highly coherent and time-dependent response after SCT.
DRG L3	38.1%	23.5%	Moderate variance explained; stable grouping and reliable biological reproducibility.
DRG L4	39.6%	32.3%	High cumulative variance (≈72%); distinct and homogeneous injury-related pattern.
DRG L5	60.3%	23.8%	Large variance captured, nearly complete separation between experimental groups, indicating pronounced injury-related molecular shifts.

In the DRG (L3–L5) datasets, two outliers were observed in each segment, including one recurring outlier from animal Hb14 (control group, Fig. 3). Technical notes indicate digestion issues for these samples during digestion (including unintended prolonged exposure to room temperature), which may have contributed to their higher variability. Please note that this sample was not removed from the dataset, as it retained sufficient data quality for downstream analysis.

### Biological validation of MNs and ECs identity

#### Validation of Cell-type identity

Proteomic profiles confirmed the expected molecular identities of both motoneuron (MN) and ependymal cell (EC) isolates, validating the accuracy of laser microdissection and sample preparation.

In MN samples, detection of neuronal and motoneuron-enriched proteins such as: Cytoskeletal and axonal transport proteins: *Tubb3, Tuba1a, Nefh, Nefm, Nefl, Kif5a, Dync1h1, Map1b, Ank2;* Synaptic vesicle and neurotransmission components: *Snap25, Syt2, Syn1, Syp, Vamp1, Stx1b, Rab3a, Cplx1;* Neuron-specific RNA-binding proteins: *Elavl3 (HuC), Rbfox3 (NeuN), Nova1*. These neuronal markers confirm the integrity of the motoneuron proteome and validate successful retrograde labeling and laser microdissection.

In ECs isolates, proteomic signatures were consistent with the epithelial phenotype of the spinal central canal lining. Strong expression of junctional and desmosomal proteins—*jup*, *dsp*, *dsg1*, and *ctnna1*—confirmed preserved cell-cell adhesion complexes typical of the ependymal barrier. The detection of *sparcl1* and *dclk1* supported an ependymal and radial-glia-like identity, while mitochondrial proteins (*tomm34*, *timm44*, *ndufa12*) reflected the high metabolic activity characteristic of this epithelium. The presence of *s100a13* and *ro60*, associated with epithelial stress and remodeling, aligned with post-injury activation of the central canal region.

#### Limitations and cellular overlap

Although the MNs proteomes show strong neuronal specificity, several canonical cholinergic markers expected in MNs — including *chat* (choline acetyltransferase) and *slc18a3* (vesicular acetylcholine transporter, VAChT) — were not detected. This likely reflects the low abundance of these proteins in microdissected cell bodies, as these proteins are mostly accumulated in the axonal compartment and therefore are underrepresented in LC-MS/MS datasets. Likewise, muscarinic receptors (*chrm2*, *chrm4*) were absent, consistent with poor recovery of GPCRs in standard proteomic workflows. Low-level detection of astrocytic proteins such as *gfap* and *aldh1l1* likely results from perisomatic glial processes that are difficult to exclude during microdissection. Overall, MN datasets are neuronally dominated and biologically consistent, though membrane receptor coverage and minor glial signals remain inherent technical limitations.

Although ECs proteomes showed strong epithelial and junctional signatures, several classical ependymal and ciliary markers (e.g., *foxj1, dnaic1*) were not detected. Low detection level of glial and extracellular matrix proteins probably reflects partial inclusion of periependymal tissue during microdissection.

A limitation of this study is the exclusive use of male rats, chosen to maintain continuity with our previous datasets and ensure comparability across studies. Given reported sex differences in spinal cord injury responses^37–39^, future work including female animals will be necessary to evaluate potential sex-specific proteomic changes.

## Data Availability

The data deposited online via the PRIDE partner repository with the dataset identifier PXD070428 was supplied as raw LC-MS/MS files and result files from MaxQuant. The raw files can be used for additional searches (e.g. with different parameters, different software). The results of MaxQuant searches can be further processed (filtered, normalized, analyzed statistically, visualized, etc.). Alternatively, tables with processed data and comparisons between sample groups were deposited at RepOD (Open Data Repository, 10.18150/9VXNRH).

## Code availability

No custom code has been used.
